# Sialic Acid Derivatization of Fluorescently Labeled *N*-Glycans Allows Linkage Differentiation by Reversed-Phase Liquid Chromatography–Fluorescence Detection–Mass Spectrometry

**DOI:** 10.1021/acs.analchem.1c02610

**Published:** 2022-04-28

**Authors:** Alan B. Moran, Richard A. Gardner, Manfred Wuhrer, Guinevere S. M. Lageveen-Kammeijer, Daniel I. R. Spencer

**Affiliations:** † Center for Proteomics and Metabolomics, Leiden University Medical Center, 2300 RC Leiden, The Netherlands; ‡ Ludger Ltd., Culham Science Centre, OX14 3EB Abingdon, United Kingdom

## Abstract

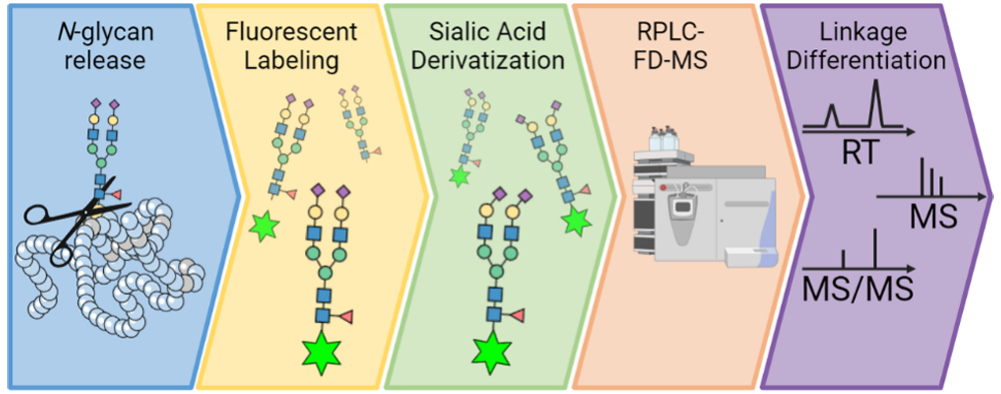

Sialic acids have diverse biological roles, ranging from promoting up to preventing protein and cellular recognition in health and disease. The various functions of these monosaccharides are owed, in part, to linkage variants, and as a result, linkage-specific analysis of sialic acids is an important aspect of glycomic studies. This has been addressed by derivatization strategies using matrix-assisted laser desorption/ionization mass spectrometry (MS) or sialidase digestion arrays followed by liquid chromatography (LC)-MS. Despite this, these approaches are unable to simultaneously provide unambiguous assignment of sialic acid linkages and assess further isomeric glycan features within a single measurement. Thus, for the first time, we present the combination of procainamide fluorescent labeling with sialic acid linkage-specific derivatization via ethyl esterification and amidation for the analysis of released plasma *N*-glycans using reversed-phase (RP)LC-fluorescence detection (FD)-MS. As a result, α2,3- and α2,6-sialylated *N*-glycans, with the same mass prior to derivatization, are differentiated based on retention time, precursor mass, and fragmentation spectra, and additional sialylated isomers were also separated. Furthermore, improved glycan coverage and protocol precision were found via the novel application using a combined FD-MS quantification approach. Overall, this platform achieved unambiguous assignment of *N*-glycan sialic acid linkages within a single RPLC-FD-MS measurement, and by improving their retention on RPLC, this technique can be used for future investigations of released *N*-glycans as an additional or orthogonal method to current analytical approaches.

## Introduction

Sialic acids are important monosaccharides that play a role in a wide range of biological processes.^1^ Often found as the terminal residue on *N*- and *O*-linked glycans as well as glycolipids,^2^ sialic acids act as mediators during biological recognition.^1^ This includes processes such as protein and cell binding as well as host–pathogen interactions.^3^ Another important role for sialic acids is their masking effect.^4^ For example, α2,3-linked sialic acids allow the underlying galactose to be accessed by specific lectins, whereas α2,6-linked sialic acids may act as inhibitors of such processes.^5^ These are important functions during numerous healthy and disease states, including cancer metastasis and tumor cell survival.^2^

Due to their critical role in biology, linkage-specific analysis of sialic acids is an important facet of glycomic investigations. However, the labile nature and negative charge of these monosaccharides presents several challenges for mass spectrometry (MS)-based analyses.^6^ Matrix-assisted laser desorption/ionization mass spectrometry (MALDI)-MS approaches have largely overcome these issues by employing linkage-specific sialic acid derivatization,^7,8^ which has the effect of stabilizing sialic acids and neutralizing their charge to ensure a more homogeneous ionization.^8^ Despite this, MALDI-MS methods generally lack an online separation component which is useful to assess further structural aspects that may differentiate glycan isomers. For this purpose, liquid chromatography (LC)-MS techniques, such as porous graphitized carbon (PGC)-LC, reversed-phase (RP)-LC, and hydrophilic interaction-LC (HILIC), are often more suitable approaches. PGC-LC is a powerful approach for in-depth structural differentiation of glycans,^9^ however, it is applied in only a few laboratories due to its complexity.^9,10^ Although RPLC is a more widely used technique, it is often insufficient to separate several glycan species, particularly sialylated *N*-glycans, as the separation of *N*-glycans is largely influenced by the reducing-end label.^11^ As a result, HILIC is most often the LC method of choice for released *N*-glycan analysis.^12,13^ Despite this, unequivocal sialic acid linkage assignment may not be achieved within a single measurement and further analysis using sialidase enzymes is required.^14^

Unambiguous linkage assignment and online separation were achieved when sialic acid derivatization was combined with reducing-end fluorescent labeling using 2-aminobenzamide (2-AB).^15,16^ This allowed measurement by HILIC-MS while also producing linkage-specific mass shifts and fragmentation spectra. However, the DMT-MM (4-(4,6-Dimethoxy-1,3,5-triazin-2-yl)-4-methyl morpholinium chloride) derivatization procedure involves harsher reaction conditions in comparison with more recently developed protocols.^6,17^ In addition, the nature of such chemical modifications to sialic acids increases their hydrophobicity and reduces their retention time when analyzed using HILIC.^15^ This is problematic for the analysis of complex samples, particularly when using fluorescence detection, as a diverse range of glycan species elute along the entire profile.^18^ In this regard, it may be more suitable to analyze glycans with enhanced hydrophobicity by RPLC. This was previously demonstrated on RPLC-MS using 2-aminopyridine (2-PA) or Girard’s P reagent labeled glycans, in combination with sialic acid linkage-specific derivatization via two-step alkylamidation or deuterated aniline amidation, respectively.^19,20^ Interestingly, both of these studies employed a charge-based fractionation step prior to sialic acid derivatization that allowed in-depth structural characterization to be performed specifically on sialylated fractions. Despite this, the fractionation employed in these studies results in the loss of information regarding nonsialylated species, and may hinder the analysis of large numbers of samples as well as result in sample loss.

This study aimed to develop and validate a platform for sialic acid-linkage specific differentiation of fluorescently labeled released *N*-glycans from a complex sample. As a result, ethyl esterification and amidation (EEA) was performed on procainamide-labeled *N*-glycans from plasma, which allowed the *N*-glycans to be effectively analyzed using RPLC-FD-MS. In addition, the method was developed on a robot which allowed it to be qualified using a large number of replicates (*n* = 50). Finally, this research also sought to explore the complementarity of the newly developed EEA and RPLC-FD-MS platform with the current gold-standard method for released *N*-glycan analysis, HILIC-FD-MS.

## Experimental Section

### Materials

Lyophilized human plasma [P9523] (5 mL), formic acid (FA), methanol, hydrate 1-hydroxybenzotriazole (HOBt), and ammonia (28% NH_3_) were purchased from Sigma-Aldrich (Dorset, UK). Acetonitrile (CH_3_CN; Romil, 190 SPS for UV/gradient quality) and ethanol (EtOH) were acquired from Charlton Scientific (Charlton, UK). Deionized water (H_2_O) was obtained using a Sartorius arium comfort (Goettingen, Germany) with 18.2 MΩ resistivity and 1-ethyl-3-(3-(dimethylamino) propyl) carbodiimide (EDC) was purchased from Fluorochem (Hadfield, UK). PNGase F storage buffer, composed of 50 mM sodium chloride (NaCl), 5 mM ethylenediaminetetraacetic acid (EDTA), and 20 mM tris-hydrochloric acid (Tris-HCl, pH 7.5), was purchased from New England Biolabs (Hitchin, UK). *N*-glycan A2G2S2 standard [CN-A2–20U], the PNGase F *N*-Glycan release kit [LZ-rPNGASEF-96], Protein Binding Membrane (PBM) plate [LC-PBM-96], 2 mL 96-well collection plate [LP-COLLPLATE-2 ML-96], procainamide labeling kit [LT-KPROC-96], HILIC cleanup plate [LC-PROC-96] and ammonium formate solution [LS-N-BUFFX40] were purchased from Ludger Ltd. (Abingdon, UK). The 120 μL skirted 96-well PCR plate [4ti-0960/C], 300 μL nonskirted 96-well PCR plate [4ti-0710/C], 1.2 mL 96 well deepwell plate, foil pierce seal [4ti-0531], and peel seal [4ti-0521] were purchased from 4titude Ltd., (Surrey, UK). HPLC vials [186002639] were purchased from Waters Ltd., (Borehamwood, UK). The human milk oligosaccharide standards, sialyllacto-N-tetraose c (LST-C) [SLN506] and sialyllacto-N-tetraose a (LST-A) [SLN503], were purchased from Dextra (Reading, UK).

### *N*-Glycan Analysis

Commercial lyophilized human plasma was reconstituted in 5 mL H_2_O, at a final concentration of 1 mg/mL. Preparation of plasma *N*-glycans was carried out in line with previously published procedures using a Hamilton Microlab STARlet liquid-handling robot.^21^ The experimental procedures for performing PNGase F *N*-glycan release and procainamide labeling are included in Supporting Information 1, sections S1.1 and S1.2.

### Sialic Acid Ethyl Esterification and Amidation

The lyophilized released and procainamide-labeled *N*-glycan samples were reconstituted in 15 μL H_2_O. The ethyl esterification and amidation (EEA) protocol was performed as previously described^17^ and was automated on the Hamilton Starlet. Briefly, the ethyl esterification reagent was prepared (250 mM EDC and 250 mM HOBt dissolved in EtOH) and 60 μL was added per well in a 300 μL 96-well PCR plate. Following this, 3 μL of the concentrated procainamide-labeled *N*-glycans was added to the reagent, then the plate was sealed with a foil pierce seal and incubated for 60 min at 37 °C. Following this, 12 μL of 28% NH_3_ was added to the samples before the plate was resealed and incubated for another 60 min at 37 °C. A volume of 225 μL CH_3_CN was added to the plate bringing the final volume in each sample well up to 300 μL. A HILIC cleanup plate was placed on the vacuum manifold and prepared with successive washes of 200 μL of 70% EtOH/H_2_O (*v/v*), 200 μL of H_2_O and 200 μL of CH_3_CN. Then, 100 μL CH_3_CN was added to each well of the cleanup plate followed by 100 μL of the derivatized and labeled sample. The samples were eluted under gravity for 5 min before a vacuum was applied. This step was repeated two more times until the entire 300 μL of the derivatized and labeled sample was transferred to the cleanup plate. The plate was blotted briefly onto a paper towel in order to remove excess CH_3_CN before being placed back on the vacuum manifold. Following this, a 96-well 2 mL collection plate was placed inside the vacuum manifold and 100 μL H_2_O was added to the samples. To start the sample elution a vacuum was used for about 5 s, followed by further elution under gravity. After 15 min, a vacuum was applied to elute the entire sample into the collection plate. This step was repeated in order to elute the samples in a final volume of 200 μL. The remaining concentrated sample (12 μL) and the derivatized procainamide-labeled samples were stored at −20 °C until further analysis.

### RPLC-FD-MS

Samples for RPLC-FD-MS were prepared by adding 95 μL of the derivatized procainamide-labeled *N*-glycans and 5 μL CH_3_CN (5%) to a 1.2 mL deepwell plate and injecting 20 μL onto an Ultimate 3000 UHPLC system (Thermo Scientific, Hampshire, UK). An ACE excel 2 C18-PFP, 150 × 2.1 mm column (ACE Ltd., Aberdeen, UK) was used and the column temperature was set to 60 °C. The fluorescence detector (λ_ex_ = 310 nm λ_em_ = 370 nm) sensitivity was set to 8 and bulb power was set to “high”. A separation gradient was employed using solvent A (50 mM ammonium formate) and solvent B (10% CH_3_CN; 0.1% FA (*v/v*)): 0 to 26.5 min, 15 to 95% solvent B; 26.5 to 30.5 min, 95% B; 30.5 to 32.5 min, 95 to 15% B; 32.5 to 35.1 min, 15% B, all gradient steps were performed with a flow rate of 0.4 mL/min.

MS analysis was performed via coupling the UHPLC to an amaZon Speed ETD MS (Bruker Daltonics GmbH, Bremen, Germany) using electrospray ionization (ESI). The instrument was operated in positive ionization mode with enhanced resolution scanning. The mass range (*m*/*z* 600–1600) was scanned with a target mass set to *m*/*z* 900. In addition, the following parameters were employed: source temperature 250 °C; gas flow 10 L/min; capillary voltage 4500 V; ion charge control (ICC) target 200,000; max accumulation time 50.00 ms. Furthermore, MS/MS spectra were obtained via collision-induced dissociation according to these conditions: ICC target MS(n) 200,000; max accumulation time MS(n) 40.00 ms; number of precursors ions selected 3; release after 0.2 min; MS(n) scan range selection, scale to precursor; absolute and relative signal threshold for automatic MS(n), 25,000 and 5%, respectively. Information regarding HILIC-FD-MS measurements and the intermediate precision and repeatability study may be found in Supporting Information 1, sections S1.3 and S1.4, respectively.

### *N*-Glycan Assignments

RPLC peaks were screened using the Bruker DataAnalysis software (version 5.0) and structures were manually assigned. Assignments of structures were made based on their exact mass, fragmentation pattern, and retention order. Important diagnostic ions for assignment are included by Supporting Information 2, Table S1, as well as the analysis and scan number of MS/MS spectra. Furthermore, a comprehensive overview of *N*-glycans reported across several studies in plasma has previously been published by Lageveen-Kammeijer et al.^17^ Thus, structures that were assigned in our study were compared against the plasma *N*-glycans present in this overview. HILIC peaks were assigned in conjunction with elution positions reported in the literature^13^ as well as by searching the GlycomeDB database using Bruker Proteinscape (version 4.0). In this instance, the database search was narrowed by employing the following parameters: assignment score (≥30), *N*-glycan fragment coverage (≥20%), CID classification depth (≥3), and accurate mass (±100 ppm). *N*-glycan compositions are illustrated according to the Consortium for Functional Glycomics (CFG) notation:^22^*N*-acetylglucosamine (N; blue square), fucose (F; red triangle), galactose (H; yellow circle), mannose (H; green circle), *N*-acetylneuraminic acid (S; purple diamond).

### Fluorescence Detection-Mass Spectrometry (FD-MS) Quantification

After FD and MS curation, as described in Supporting Information 1, sections S1.5 and S1.6, respectively, only the fluorescent peaks that contained at least one *N*-glycan that passed were considered for further analysis. This was followed by determining the proportion of *N*-glycan compositions in each fluorescent peak by calculating the local relative abundance of compositions eluting under the same chromatographic peak using MS signal intensities. Following this, the FD-MS signal was derived by multiplying the proportion of each *N*-glycan composition by the fluorescent signal of the peak in which it is eluting.

## Results and Discussion

**Figure 1:**
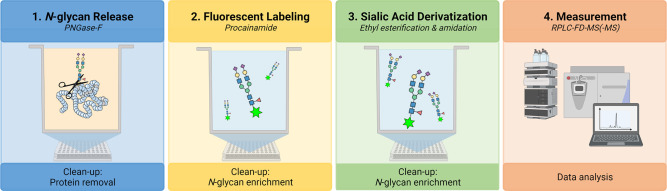
Semi-automated workflow for the *N*-glycan release, procainamide labeling, sialic acid derivatization, and RPLC-FD-MS measurement. The workflow was completed on a Hamilton Microlab STARlet liquid-handling robot allowing 96 samples to be processed simultaneously. *N*-glycans were released from plasma proteins during an overnight digestion (37 °C). Following protein removal, *N*-glycans were fluorescently labeled using procainamide and enriched via a HILIC cleanup plate. Sialic acids were derivatized by ethyl esterification and amidation, and enrichment was repeated using the HILIC cleanup plate. Fluorescently labeled and derivatized sialic acid *N*-glycans were measured using RPLC-FD-MS.

For the first time, we present the combination of procainamide fluorescent labeling with a sialic acid linkage-specific derivatization step via EEA for the analysis of *N*-glycans by RPLC-FD-MS (Figure 1). Procainamide was selected as the fluorescent label of choice because it is a well-established amination reagent, and EEA was selected as the derivatization strategy as it has been widely applied, is well-developed,^17,23^ may be performed under relatively mild conditions, and promotes the formation of stable sialic acid derivatives during the reaction.^6,23^ Several parameters were investigated in order to develop the protocol, which are summarized in Supporting Information 1, section S2.1 and Supporting Information 2, Table S2. In addition, the intermediate precision and repeatability of the complete sample preparation protocol was determined and the separation of the EEA-derivatized *N*-glycans on RPLC was evaluated, as well as several quantification approaches. Finally, the complementarity of RPLC- and HILIC-FD-MS platforms was also assessed.

### Sialic Acid Differentiation by RPLC-FD-MS

**Figure 2:**
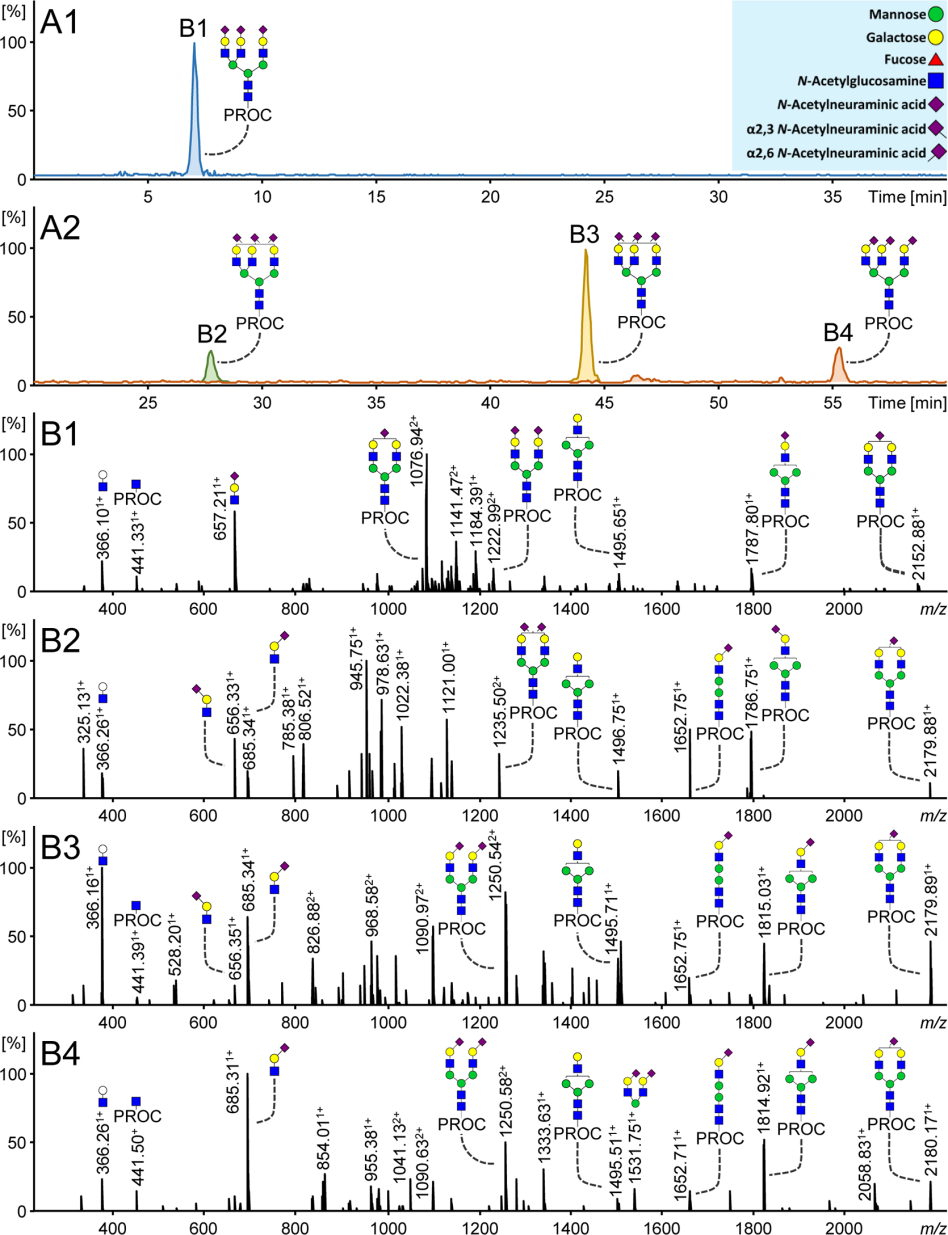
Procainamide-labeled trisialylated plasma *N-*glycans measured by RPLC-FD-MS. (A1) H6N5S3 (*m*/*z* 1033.73) with procainamide labeling and without sialic acid linkage-specific derivatization. (A2) H6N5S3 is separated into three distinct isomers following procainamide labeling and sialic acid linkage-specific derivatization: H6N5S_2,3_2S_2,6_1, H6N5S_2,3_1S_2,6_2, and H6N5S_2,6_3 (*m*/*z* 1042.42, 1052.09, and 1061.77, respectively). Panels B1–B4 show the corresponding MS/MS spectra. Specific ions such as B-ions 656.25 and 685.26 confirm the type of sialic acid linkage(s) present. In the case of multiple charge states of a single fragment, only one charge state is annotated, such as *m*/*z* 2179.89 [M + H]^1+^ (*m*/*z* 1090.97 [M+2H]^2+^) and *m*/*z* 1652.75 [M + H]^1+^ (*m*/*z* 826.88 [M+2H]^2+^). Monosaccharide annotation is provided in the blue box.

Procainamide-labeled sialylated *N*-glycans showed short elution times on RPLC and no separation of sialic acid linkage isomers. An example is provided in Figure 2A1, where the *N*-glycan, H6N5S3, elutes at 7 min as a single peak. Based on the RPLC separation, there is no evidence to suggest that this analyte consists of multiple linkage species. In general, the RPLC profile of procainamide-labeled *N*-glycans (Supporting Information 1, Figure S1A1) showed an elution pattern similar to 2-AB and 2-AA labeled *N*-glycans whereby sialylated structures are poorly separated.^24^ In comparison, labels such as 2-PA^25^ or Rapifluor-MS^26^ generally demonstrated an enhanced separation with sialylated species eluting according to an increasing number of sialic acid residues. Interestingly, isomer separation of procainamide-labeled glycans was detected using RPLC for glycans with incomplete antenna sialylation, as shown by H5N4S1 in Supporting Information 1, Figure S1A2. This is consistent with findings obtained with the aforementioned fluorescent labels.^24−26^ With regard to this, studies have described the different contribution of antennae to the retention time on RPLC.^25,27^ Thus, likely positional isomers are separated based upon which arm is occupied (α3 versus α6). Overall, procainamide has demonstrated enhanced fluorescence in comparison with 2-AB,^28^ 2-AA, and 2-PA, as well as a greater positive ionization (MALDI and ESI) response than 2-AB^28^ and 2-AA.^29^ In addition, although RapiFluor-MS allows faster sample preparation and has shown increased ionization efficiency,^26^ procainamide offers the advantage of being a widely available chemical that may be purchased in bulk or as part of specific glycan labeling kits.

In order to enable the separation of fluorescently labeled sialylated *N*-glycans on RPLC, EEA was employed prior to measurement in order to enhance glycan hydrophobicity. The derivatization of sialic acids (Figure 2A2) not only improves their retention, but also allows the resolution of three distinct sialic acid-linkage isomers, H6N5S_2,3_2S_2,6_1, H6N5S_2,3_1S_2,6_2, and H6N5S_2,6_3, because of the different chemical derivatization of differently linked sialic acids. Notably, Figure 2 also shows that the position of the sialic acid on the galactose remains ambiguous and further topological isomers may exist for the trisialylated species with mixed linkages at Figure 2B2,B3. Importantly, ethyl esterified α2,6-sialylated species showed greater retention and were separated from their amidated α2,3-sialic acid counterparts in order of increasing α2,6-sialic acid content. Additionally, EEA differently modifies the mass of sialylated *N*-glycans depending on the linkage and number of sialic acids present: α2,6-linked sialic acids gain 28.02 Da, and α2,3-linked sialic acids lose 0.98 Da.^17^ This is illustrated in Figure 2A1 whereby *m*/*z* 1033.73 is used to generate an extracted ion chromatogram (EIC) for H6N5S3 [M+2H]^2+^, whereas three distinct precursor *m*/*z* values are used for each isomer in order to generate EICs ([M+2H]^2+^) in Figure 2A2: *m*/*z* 1042.42 (H6N5S_2,3_2S_2,6_1), 1052.09 (H6N5S_2,3_1S_2,6_2), and 1061.77 (H6N5S_2,6_3). Previously, similar improvements to RPLC separation and sialic acid linkage differentiation have also been shown using other labeling and derivatization techniques.^19,20^ Overall, these results suggest that the performance of fluorescently labeled glycans on RPLC may be generally enhanced by including a sialic acid derivatization step because the combination of these techniques is required for efficient separation and differentiation on RPLC.

The linkage assignment of derivatized sialylated *N*-glycans can be further supported by diagnostic ions in the MS/MS spectra. In contrast with Figure 2B1 whereby *m*/*z* 657.21^1+^ indicates a sialylated antenna with unspecified linkage, panels B2–B4 in Figure 2 show informative B-ions with theoretical *m*/*z* 656.25^1+^ and 685.26^1+^, which indicate amidated α2,3- and esterified α2,6-antennae, respectively. In addition, several Y-ions provide further support for the assignments. For example, Figure 2B2 shows a fragment at *m*/*z* 1787.80^1+^, indicating the composition H5N4S_2,3_1, resulting from the loss of two sialylated antennae. A similar fragment at *m*/*z* 1815.03^1+^ with an α2,6-sialic acid is shown in Figure 2B3. Another B-ion with two α2,6-linked sialylated antennae attached to a mannose is detected at *m*/*z* 1531.75^1+^ in Figure 2B4. Furthermore, no ions corresponding to α2,3-linked sialic acids are detected in Figure 2B4 as the *N*-glycan solely contains α2,6-sialylated species. Thus, consistent with previous studies that employed sialic acid linkage-specific derivatization,^8,17,23^ the present method allows the unambiguous assignment of the sialic acid linkages present in plasma *N*-glycans using RPLC-MS.

Interestingly, Figure 2 illustrates a greater relative abundance of the derivatized sialylated oxonium ions; in particular, *m*/*z* 685.31^1+^ (Figure 2B4) shows a 100% relative abundance for the *N*-glycan that is fully occupied with α2,6-linked sialic acids. This is in comparison with the nonderivatized ion *m*/*z* 657.21^1+^ (60%; Figure 2B1). Furthermore, derivatized Y-ions such as *m*/*z* 1235.50^2+^ and 1250.54^2+^ in panels B2 and B3 of Figure 2, respectively, also appear to have a greater relative abundance than their nonderivatized counterpart *m*/*z* 1222.99^2+^ (Figure 2B1). Similar results were also obtained when Suzuki et al. compared nonderivatized and derivatized glycans from human α-1-acid glycoprotein (α1-AGP).^19^ Thus, this is likely due to the greater stability that derivatized sialic acids exhibit during fragmentation following derivatization. However, an improvement in ionization efficiencies due to derivatization can also not be discounted. Nonetheless, the increase in relative abundance of fragment ions in the MS/MS spectra greatly enhances the identification of glycan structures. Further studies should focus on comparing derivatized and nonderivatized complex glycan standards while controlling for the injection amounts in order to explore this phenomenon further.

Separation of positional linkage isomers was achieved on RPLC following the combination of the procainamide labeling and EEA derivatization approaches. This is illustrated in Supporting Information 1, Figure S1B2 whereby two positional isomers are detected for each of the α2,3 and α2,6-linkage variants of H5N4S1. A comparison of the sum of the relative abundance of all isomers for H5N4S1 in the nonderivatized and derivatized profiles is provided in Supporting Information 1, Figure S1C3. It is notable that a similar total relative abundance for H5N4S1 was achieved for both profiles. This indicates that the positional isomers, determined in the nonderivatized chromatogram, were also captured in the derivatized profile, yet more information is achieved with the latter as it resolves both antenna occupation and sialic acid linkage. Interestingly, the separation time between H5N4S1 isomers in Supporting Information 1, Figure S1A2 is approximately 3 min, whereas Supporting Information 2, Figure S1B2 shows that there is a greater separation time (7 min) between H5N4S1 positional isomers with the α2,6 linkage. However, this was less apparent for positional isomers with the α2,3-linkage linkage, further indicating the different influence that the ethyl esterification and amidation modifications have in RPLC. Additionally, Supporting Information 1, Figure S2A shows that two isomers of H5N4S_2,3_1S_2,6_1 were detected. Moreover, this phenomenon has also been observed in previous studies.^19,20^ Suzuki et al. demonstrated that triantennary glycan isomers were resolved based on the attachment position of *N*-acetyl- and *N*-glycolylneuraminic acids, with these monosaccharides being attached to either galactose or *N*-acetylglucosamine (GlcNAc) residues in fetuin.^19^ However, similar to Jin et al.,^20^ here we observed that structural isomers likely containing the same sialic acid linkage(s) attached to a galactose are separated based upon which arm is occupied (α3 versus α6). Despite this, no conclusions could be drawn from the RT as well as MS/MS spectra (Supporting Information 1, Figure S2B,C) regarding which antenna occupancy gives rise to the later or earlier elution. Therefore, further research is required using well-defined standards in order to characterize this feature. Importantly, isomer separation of sialylated glycan structures has not previously been recognized as a strength of RPLC.^18^ However, the results achieved by this investigation, among others, show that both positional and sialic acid linkage isomers may be determined through the combined effect of fluorescent labeling and sialic acid derivatization.

### Plasma *N*-Glycan RPLC Profile

**Figure 3:**
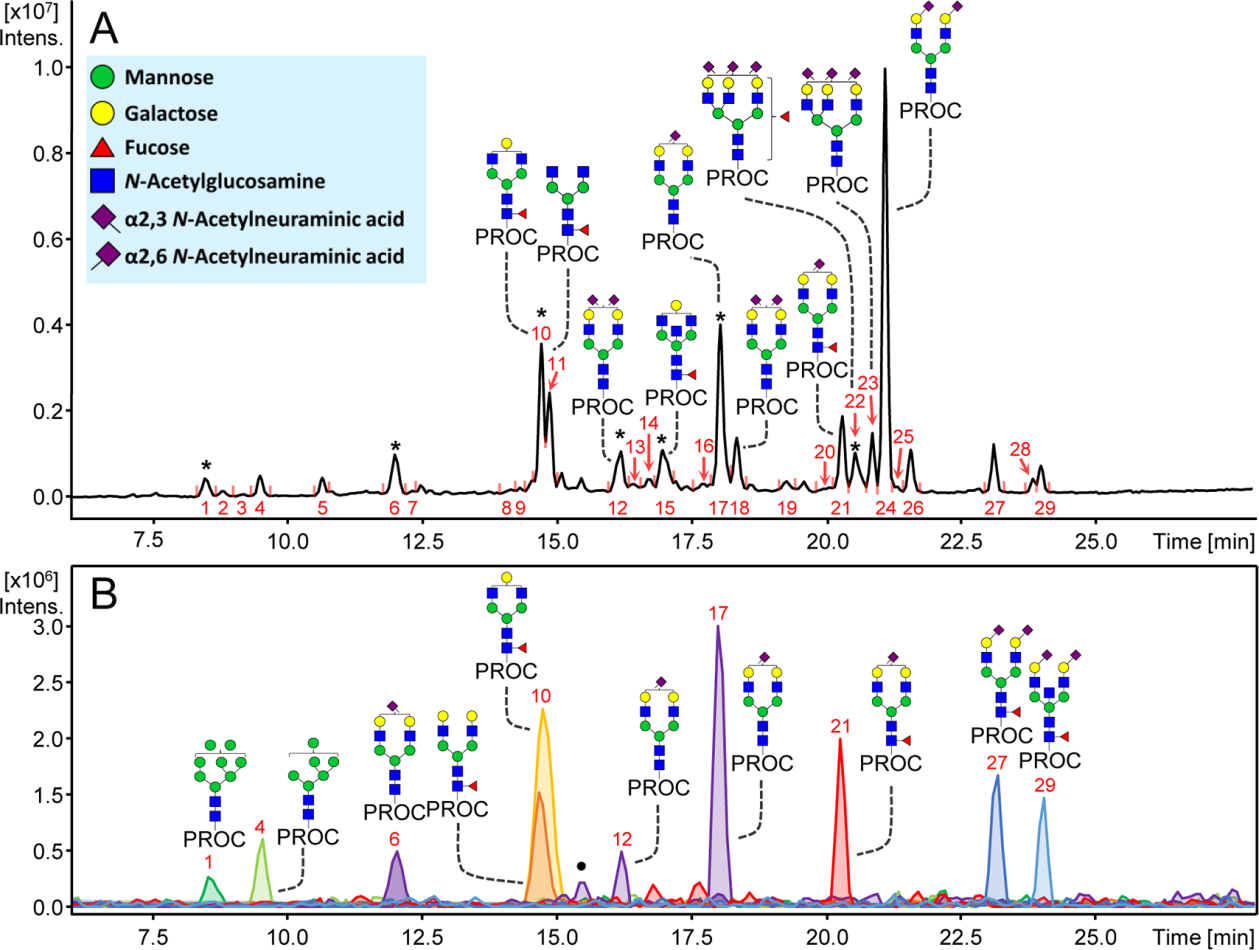
Released *N*-glycans from plasma on RPLC following procainamide fluorescent labeling and sialic acid derivatization. (A) *N*-glycan assignments of the 10 most abundant peaks are shown. In cases where multiple *N*-glycans elute under the same peak, the most abundant *N*-glycan is represented. Asterisk (*) represents peaks with multiple eluting *N*-glycans. (B) The effect of monosaccharides on RT is illustrated. This is highlighted by extracted ion chromatogram of specific *N*-glycans (from left to right): H8N2, H6N2, H5N4S_2,3_1, H5N4F1, H4N4F1, H5N4S_2,6_1 (isomer 1), H5N4S_2,6_1 (isomer 2), H5N4F1S_2,6_1, H5N4F1S_2,6_2, and H5N5F1S_2,6_2. Monosaccharide annotation is provided in the blue box. The symbol (•) denotes overlapping *m*/*z* of H5N4S_2,6_1 with a nonassigned analyte with the same *m*/*z*. Peak numbers are illustrated in red. For the full list of assignments see Supporting Information 2, Table S3.

Multiple *N*-glycan structures, including sialylated and nonsialylated structures, were resolved on RPLC following procainamide labeling and EEA. In Figure 3A, 29 fluorescent peaks were determined after data curation and the 10 most abundant peaks were assigned with the most abundant *N*-glycan based on MS detection. While some peaks are clearly dominated by a certain glycan composition, others feature several coeluting *N*-glycans (Supporting Information 2, Table S3). The specific influence of different monosaccharides on retention is highlighted in Figure 3B. A decreasing number of mannoses in the *N*-glycan composition is associated with higher hydrophobicity.^13^ This is exemplified by the high-mannose structures that are eluting first in the profile, and the EICs of *N-*glycans H8N2 and H6N2 demonstrate that elution occurs in order of decreasing hexose (mannose) numbers. We also observed the separation of some high-mannose isomers, similar to Chen et al.^30^ This is illustrated by the composition H7N2, which is found in peaks 2 and 3 (Supporting Information 2, Table S3). Complex nonsialylated glycans also elute in order of decreasing hexose (galactose) numbers. This is shown in Figure 3A whereby H4N4F1 (peak 10) elutes before H3N4F1 (peak 11). However, it seems a similar influence on retention is observed when the antenna is partially or fully occupied with galactose as H5N4F1 coelutes with H4N4F1 in peak 10 (Figure 3B).

A diverse number of separated sialylated *N*-glycan species are shown in the plasma profile on RPLC (Figure 3A), including positional isomers and linkage variants (Figure 3B), as previously mentioned. In contrast, only a single peak was detected here for other isomeric species such as H4N4F1 (G1F), which is known to have two positional isomers in plasma.^13^ For example, Higel et al. showed that the H4N4F1 isomers may be resolved using 2-AA.^24^ Thus, the current method may be useful for the investigation of positional sialylated isomers; however, other isomers which lack sialic acids and do not contain sufficient hydrophobic differences may not be separated and remain a challenge for this analysis.

In addition to sialic acid linkage, core fucosylation has a large influence on the RT of *N*-glycans observed here. The effect of core fucosylation is demonstrated by H5N4F1S_2,6_1 (peak 21) which elutes later than H5N4S_2,6_1 (peaks 12 and 17) in Figure 3B. Furthermore, H6N5F1S_2,3_1S_2,6_2 appears to lack a diagnostic Y-ion for core-fucosylation (*m*/*z* 587.33; N1F1-Proc), suggesting that the fucose is located on the antenna of this glycan. Thus, the influence of antennary fucosylation on RT is demonstrated by H6N5F1S_2,3_1S_2,6_2 which elutes earlier than H6N5S_2,3_1S_2,6_2 (Figure 3A). This suggests that the addition of an antennary fucose decreases the RT. These results are similar to previous findings whereby it was determined that antennary fucosylation may decrease or have a negligible influence on RT,^11,27^ depending on which antennae is fucosylated. Furthermore, the analysis of 2-PA labeled and derivatized glycans from α1-AGP showed that a triantennary *N-*glycan containing the sialyl-Lewis^x^ antigen eluted before its nonfucosylated counterpart.^19^ Importantly, antennary- and core-fucosylation confer different functions in biological systems,^31,32^ and therefore, techniques that distinguish them are required.^33^

An increasing number of GlcNAc residues results in longer RTs. However, it is difficult to define the true effect of an increasing number of antennae as this is generally accompanied by increasing glycan size due to capping by a galactose and sialic acid, the latter of which has already been shown to have a large influence on retention. In addition, it should be noted that the second isomer of the *N*-glycan H5N4S_2,3_1S_2,6_1 (peak 18) elutes later than H6N5S_2,3_1S_2,6_1 (Figure 3A and Supporting Information 2, Table S3, peak 14), despite having less antennae but the same number and linkage of sialic acids. Thus, this further highlights the large influence that derivatized sialylated positional isomers have on RT. In any case, Figure 3A shows that *N*-glycans with different numbers of antennae may be separated. Furthermore, the influence of a bisecting GlcNAc is shown in Figure 3B, as H5N5F1S_2,6_2 elutes later than H5N4F1S_2,6_2 (peaks at 22.5–25.0 min). Despite this, several other likely bisected *N*-glycan species were unable to be confirmed by MS/MS (Supporting Information 2, Table S1). However, previous research using RPLC has shown the separation of *N*-glycans containing bisecting GlcNAc from those structures without bisection. Thus, this feature is an important aspect of this method because conventional released *N*-glycan analysis often requires exoglycosidase enzymes in order to confirm bisection, and it should be further explored.

One of the main advantages of RPLC is its wide utilization and application.^11^ As a result, it is a well-developed technique and there are a large number of applications that may be implemented. For example, separation parameters can be optimized per application because a wide variety of columns are available that vary in terms of stationary phase as well as length and particle size, and they may be obtained from various manufacturers.^11^ Furthermore, nanoflow techniques may be implemented on RPLC systems in order to achieve highly sensitive analyses.^34^ Moreover, as described here and in previous studies,^24,30^ glycan analysis by RPLC is suitable for the investigation of various glycan species, such as high-mannose isomers as well as core- and antennary-fucosylated and bisected *N-*glycans. In addition, recent advancements in RP stationary phases have also improved the separation of sialylated *N*-glycans labeled with 2-AA.^35^ Despite this, sialic acid linkage and further isomer information have remained a challenge for such applications. However, recent research has shown that RPLC-MS may now be implemented for the analysis of derivatized sialylated *N*-glycans, allowing linkage-specific and isomeric information to be obtained.^19,20^ In the case of Suzuki et al., they demonstrated an in-depth approach, employing a fractionation step followed by RPLC-MS, whereas our study utilized semi-automated sample preparation and a shorter gradient time (80 vs 35 min, respectively) in order to enable more and faster analyses. Nonetheless, the application of different labeling and derivatization techniques in both studies demonstrate the potential of this approach as the combination of fluorescent labels, derivatization strategies, and RPLC systems may be explored to further enhance glycomic studies.

### Method Validation

The performance of the EEA and RPLC-FD-MS platform was validated and compared with the gold standard method for released *N*-glycan analysis, namely, HILIC-FD-MS. Crucially, for the HILIC-FD-MS platform, *N*-glycans were subjected only to the standard *N*-glycan protocol, whereby measurement is carried out following fluorescent labeling and cleanup. In addition, three quantification approaches were tested using FD, MS, and a combination of these two approaches via FD-MS across both platforms. In Table 1, it is shown that the RPLC-FD-MS platform resulted in the assignment of 29 fluorescent peaks (FD) and 39 *N-*glycan compositions (MS). Similarly, 27 fluorescent peaks and 41 *N*-glycan compositions were detected by the HILIC-FD-MS platform. In comparison, previous research determined 117 and 167 *N*-glycans in serum and plasma by HILIC-MS^36,37^ and capillary electrophoresis (CE)-MS,^17^ respectively. Importantly, these studies employed high-resolution and high-sensitivity MS. Moreover, Lageveen-Kammeijer et al. also performed linkage-specific sialic acid derivatization prior to measurement by CE-MS, a highly sensitive technique as it operates at a nanoflow level. Nonetheless, CE-MS is still not widely available in most laboratories and often lacks repeatability (capillary to capillary), long separation times (>80 min), and expertise. It is expected that the sensitivity of the developed platform reported here could be further improved via coupling with high-sensitivity MS instruments as well as employing a nanoflow column.

**Table 1:** Performance Measures of the RPLC- and HILIC-FD-MS Platforms^a^

	RPLC						HILIC					
Feature	FD		MS		FD-MS		FD		MS		FD-MS	
FD peaks (#)	29		N/A		29		27		N/A		27	
*N*-glycans (#)	N/A		39		39		N/A		41		41	
Sialic acid linkage (*a*/*b*)	N/A		22/23^*x*^		22/23^*x*^		N/A		18/24*^y^*		18/24*^y^*	
	S.D.	RSD	S.D.	RSD	S.D.	RSD	S.D.	RSD	S.D.	RSD	S.D.	RSD
Median (top 10)	0.3%	5.2%	0.5%	12.3%	0.3%	5.4%	0.1%	1.9%	0.2%	5.7%	0.1%	1.8%
Median (total)	0.2%	7.3%	0.2%	18.6%	0.2%	11.4%	0.0%	2.7%	0.1%	6.1%	0.0%	4.4%

a Features determined by these platforms include the number (#) of FD peaks and *N*-glycan compositions. In addition, the fraction (*a*/*b*) of sialic acid-linkage detected *N*-glycans is provided (*N*-glycans with sialic acid-linkage determined/total number of sialylated *N*-glycans). Sialic acid linkages were determined directly by the EEA and RPLC-FD-MS protocol, whereas assignments for HILIC-FD-MS were made in accordance with elution positions reported in the literature.^13^ Three quantification approaches are displayed: FD and MS, or the combination of these two platforms via a third quantification approach, FD-MS. Furthermore, the inter-day variation for the 10 most abundant as well as all detected *N*-glycans is provided for all three quantification approaches of each platform. Quantification of fluorescent peaks and *N*-glycan compositions determined by MS was performed using HappyTools and LaCyTools, respectively, as detailed in Supporting Information 1, sections S1.5 and S1.6. Assignments: ^*x*^confirmed by diagnostic ions in MS/MS or ^*y*^made in accordance with the literature.^13^ For the full list of assignments, see Supporting Information 2, Tables S1, S3, and S4.

The fraction of sialic acid linkage determined structures is represented in Table 1. In this case, 23 sialylated structures were determined by the RPLC-FD-MS platform, all structures could be linkage-specified by their precursor mass, and most were also confirmed via MS/MS. In comparison, 24 sialylated *N*-glycans were determined in the HILIC-FD-MS profile and 18 could be assigned with linkage information, in accordance with elution positions reported in the literature.^13^ However, in order to provide experimental results of sialic acid linkages with the HILIC-FD-MS setup, further studies are required which involve sialidase enzyme treatments. In contrast, the combination of derivatizing the procainamide-labeled glycans using EEA followed by RPLC-FD-MS analysis allows direct and unambiguous assignments of sialic acid-linkages.

The intermediate precision and repeatability of the EEA and RPLC-FD-MS platform were obtained via intra- (*n* = 3) and interday measurements (total *n* = 50). The interday relative standard deviation (RSD) of the 10 most abundant *N*-glycans (Table 1) revealed that FD had the best performance (5.2%), followed by FD-MS (5.4%) and MS (12.3%). Table 1 also shows that similar results for the three quantification approaches were obtained for the HILIC-FD-MS platform when an intraday experiment (*n* = 1; total *n* = 3) was performed. While FD showed the highest measure of performance, it provided the lowest coverage of features. This is in contrast with MS-only, as more structures could be quantified; however, there is a less precise quantification than FD. Therefore, by combining and implementing the best of the two approaches, FD-MS resulted in an increase in coverage of specific features with a higher precision than using solely MS. This is illustrated in Table 1 whereby 29 peaks were determined by RPLC-FD, whereas RPLC-FD-MS enabled the direct quantification of 39 *N*-glycan species. Similarly, HILIC-FD detected 27 peaks while HILIC-FD-MS covered 41 *N*-glycan species. Thus, a combination of FD and MS via FD-MS quantification allowed improvements for both separation platforms in regard to *N*-glycan coverage and quantification precision.

Overall, the HILIC-FD-MS platform showed higher precision than the RPLC-FD-MS method across each of the quantification approaches. Nonetheless, with regard to the 10 most abundant *N*-glycans measured by RPLC-FD-MS, both quantification approaches incorporating FD (FD and FD-MS) showed RSDs below 10%, and employing MS-only quantification resulted in an RSD below 15%. It should be noted that an overall increase in RSDs is observed when calculated for the total number of FD peaks and *N*-glycan compositions across both platforms. However, as shown in Supporting Information 2, Table S3, low-abundance glycans display higher RSDs when determined by RPLC-FD-MS in comparison with its HILIC-FD-MS counterpart. The increase in variation of the EEA and RPLC-FD-MS setup is likely to be due to the two additional sample processing steps, including a chemical derivatization and HILIC-based cleanup. Thus, further research could focus on improving the procedure by determining whether only a single cleanup step could be performed following *N*-glycan labeling and derivatization.

### Platform Complementarity

The analysis of *N*-glycans is challenging, and normally a single method is unable to capture many of the structural differences that exist between different species. However, the implementation of orthogonal methods allows in-depth and complementary information to be obtained. In this study, we examined the complementarity between the newly developed RPLC-FD-MS platform and the gold standard method, HILIC-FD-MS. The similarities and differences between these two methods are highlighted in Supporting Information 2, Table S3. As mentioned previously, H4N4F1 was detected as a single structure by RPLC-FD-MS, whereas two isomers were determined by the HILIC-FD-MS approach. Furthermore, two isomers are shown for both H5N4S_2,6_1 and H5N4S_2,3_1S_2,6_1 when analyzed by RPLC-FD-MS, whereas single structures are determined for both of these compositions by HILIC-FD-MS. Thus, a sum of both isomers may be determined by one platform whereas the alternate method may separate the small structural differences between the isomers.

In some cases, *N*-glycans were detected by only one platform. This is shown in Supporting Information 2, Table S3 where two isomers of H6N5S_2,3_1S_2,6_1 were analyzed by RPLC-FD-MS whereas this structure was not detected by HILIC-FD-MS. Overall, 11 and 13 unique structures were quantified by RPLC-FD-MS and HILIC-FD-MS, respectively (Supporting Information 1, Figure S3A). Despite this, these unique structures account for only 5% and 8% of total areas determined by these platforms, respectively (Supporting Information 1, Figure S3B). In contrast, there are 28 *N*-glycans that were determined by both methods (Supporting Information 1, Figure S3A), covering 95% of the total area for RPLC-FD-MS and 92% of the total area for HILIC-FD-MS. This shows that both platforms cover the majority of detected *N*-glycans and, as a result, provide important orthogonal information regarding the sample.

The association between the relative abundances of *N*-glycans determined by both platforms was investigated. This was performed by examining the 28 overlapping structures only (renormalized to the sum of the total area of these compositions). In the case of isomeric species, their relative abundances were summed in order to perform the comparison between both platforms (Supporting Information 1, Figure S4). Importantly, H5N4S_2,6_1 and H5N4S_2,6_2 were not plotted as these two structures are much more abundant in plasma than other *N*-glycans and may result in an overestimation of association between the two platforms. Similar relative abundances were determined for overlapping structures (*R*^2^ = 0.78); however, some discrepancies in quantification may arise from summing isomer signals for the purposes of the comparison, as well as slight ionization biases due to differential derivatization.^38^ Nonetheless, this study shows that both platforms may be applied to the same sample and used as complementary approaches.

## Perspectives

The protocol presented here represents an important development for the application of RPLC-MS to analyze released *N*-glycans, enabling the elucidation of sialic acid linkage-specificity. Nevertheless, further developments should be carried out in order to further explore and exploit the capabilities of this technique. For example, the effects of isomers on separation should be defined using well-established *N*-glycan standards. In addition, further identification of byproducts related to labeling or derivatization should be performed. Finally, the combination of various derivatization and labeling strategies could also be explored.

## Conclusions

The developed platform allows released sialylated *N*-glycans to be efficiently analyzed using RPLC-FD-MS(/MS), and the procedure is compatible with, and complementary to, the standard *N*-glycan processing protocol. Thus, the platform is applicable where unambiguous sialic acid linkage assignment is required from a single measurement, in addition to information regarding specific types of *N*-glycan isomers. The investigation of isomeric species such as these is not a common application of RPLC techniques. Thus, this approach allows greater access to a platform that is already well-developed, widely available, and easily applicable for the linkage-specific analysis of sialylated *N*-glycans.

## Supporting Information Available

Supporting Information 1: additional experimental section (section S1); additional results section regarding method development (section S2); Supporting Information figures (section S3); Supporting Information references (section S4) (PDF)
Supporting Information 2: Supporting Information tables (XLSX)

## Notes

The data with regard to the intra- and interday validation are available using the identifier GPST000190 at the GlycoPost repository.^39^ All other data, such as that relating to the method development, are available from the corresponding author upon request.
