# Supplementary material for: Sialic Acid Derivatization of Fluorescently Labeled N-Glycans Allows Linkage Differentiation by Reversed-Phase Liquid Chromatography–Fluorescence Detection–Mass Spectrometry
Source: Anal Chem. 2022 Apr 28;94(18):6639–48. doi: 10.1021/acs.analchem.1c02610 (PMC9096788; doi:10.1021/acs.analchem.1c02610)
Supplement: Supplementary file 1 — ac1c02610_si_001.pdf [file ac1c02610_si_001.pdf]

Supporting Information

Sialic acid derivatization of fluorescently labeled *N*-glycans allows linkage differentiation by RPLC-FD-MS

Alan B. Moran[1,2]*, Richard A. Gardner[2], Manfred Wuhrer[1], Guinevere S.M. Lageveen-Kammeijer[1], Daniel I.R. Spencer[2,]

1 Leiden University Medical Center, Center for Proteomics and Metabolomics, 2300 RC Leiden, The Netherlands

2 Ludger Ltd., Culham Science Centre, OX14 3EB Abingdon, United Kingdom

* **Correspondence**: Alan Moran, Leiden University Medical Center, Center for Proteomics and Metabolomics, P.O. Box 9600, 2300 RC Leiden, The Netherlands; a.b.moran@lumc.nl; Tel: +31-71-5266982

Table of Contents

S1. Supporting Information - Experimental Section S3

S1.1 PNGase F release S3

S1.2 *N*-glycan fluorescent labeling S4

S1.3 HILIC-FD-MS S5

S1.4 Intermediate precision and repeatability S5

S1.5 Fluorescence detection (FD) data processing S5

S1.6 MS data processing S6

S2. Supporting Information - Results S7

S2.1 Method development S7

S3. Supporting Information – Figures S9

Figure S1. Non-derivatized versus derivatized procainamide-labeled plasma *N*-glycans on RPLC (70 min gradient) S9

Figure S2. Sialic acid linkage-specific MS and MS/MS spectra S10

Figure S3. Unique and overlapping *N*-glycan features detected by the RPLC-FD-MS and HILIC-FD-MS platforms S11

Figure S4. Comparison of relative abundances of 26 overlapping *N*-glycans detected between the RPLC-FD-MS and HILIC-FD-MS platforms. S12

Figure S5. Adduct ion formation during MS measurement S13

Figure S6. Derivatized and procainamide-labeled plasma *N*-glycan profile on RPLC S14

S4. References S15

S1. Supporting Information - Experimental Section

S1.1 PNGase F release

Plasma (5 µL) and H_2O (4 µL) were added to a 120 µL 96-well PCR plate. In addition, 10X denaturing solution (1 µL), consisting of 5% sodium dodecyl sulfate with 400 mM dithiothreitol [LZ-rPNGASEF-96], was added to the samples and the plate was sealed with a foil pierce seal. The plate was briefly vortexed and centrifuged prior to a 10 min incubation at 100 °C. The plate was left to cool to room temperature and the foil pierce seal was removed. Following this, 6 µL of H_2O, 2 µL of concentrated reaction buffer (500 mM sodium phosphate, pH 7.5), and 2 µL 10% NP-40 were added to each sample. The PNGase F solution (150 µL) from the kit was diluted with 150 µL PNGase F storage buffer and 2 µL was added to each sample. The plate was sealed using a foil pierce seal and incubated overnight at 37 °C. Following the incubation, the foil pierce seal was removed and 5 µL of a 5% FA/H_2O solution (*v/v*) was added to each sample. The plate was sealed again, vortexed to mix, briefly centrifuged and incubated at room temperature for 40 min. In order to remove the enzyme and other protein contaminants, a PBM plate was set up on the vacuum manifold on the Hamilton robot. The plate was prepared by washing each well first with 100 µL of methanol, followed by 300 µL of H_2O. The solutions were removed by applying a vacuum prior to adding the next solution. The samples in the PCR plate (27 µL) were added to the PBM plate and the PCR plate was washed with 90 µL H_2O, which was added to the corresponding sample well. Following this, the PBM plate was placed on top of a 2 mL collection plate and centrifuged using a Heraeus Labofuge 400 R (800 rcf for 3 min, 22°C). Finally, 100 µL H_2O was added to the PBM plate and elution via centrifugation was repeated. The samples were transferred to a 300 µL 96-well PCR plate and dried down using a Thermo Savant vacuum centrifuge.

S1.2 *N*-glycan fluorescent labeling

Plasma samples were procainamide-labeled in the PCR plate using the Hamilton robot whereas the *N*-glycan and human milk oligosaccharide standards were prepared manually, as previously described.[1] In order to prepare the procainamide labeling kit for the Hamilton robot-based protocol, 1.2 mL of 30% acetic acid in dimethyl sulfoxide from the kit and 1.2 mL H_2O were added to the procainamide dye followed by vortexing to ensure the dye was dissolved. This procainamide solution was added to the sodium cyanoborohydride reductant and vortexed again to mix and dissolve. The solution was added to two 2 mL Sarstedt vials that were placed on the robot deck, which added 20 µL of the labeling solution to each sample. Following this, the 300 µL 96-well PCR plate was manually sealed with a pierce-foil seal. The plate was briefly vortexed and centrifuged followed by incubating the samples for 60 min at 65 °C.

A HILIC clean-up plate was used to remove an excess of the labeling reagent. The clean-up plate was placed on the vacuum manifold on the Hamilton robot and successive washes with 200 µL of 70% EtOH/ H_2O (*v/v*), 200 µL of H_2O and 200 µL of CH_3CN were performed. To each sample CH_3CN (230 µL) was added, aspirated and dispensed five times to mix after being transferred to the clean-up plate. The samples were eluted under gravity for 10 min before a vacuum was applied. The samples were then washed with 200 µL of CH_3CN and elution was performed under gravity for 5 min before a vacuum was applied. This step was performed two more times. The clean-up plate was blotted briefly onto a paper towel in order to remove excess CH_3CN before being placed back on the vacuum manifold. Following this, a 96-well 2 mL collection plate was placed inside the vacuum manifold and 100 µL H_2O was added to the samples. To start the sample elution a vacuum was used for about 5 sec, followed by elution under gravity for 15 min before a vacuum was applied again to elute any remaining sample. This step was repeated in order to elute the released and labeled *N*-glycans in a final volume of 200 µL. Following this, 120 µL of sample was transferred to a 300 µL 96-well PCR plate and reduced to dryness using a vacuum centrifuge. The 96-well 2 mL collection plate which contained the remaining 80 µL of sample was stored at -20 °C prior to HILIC-MS analysis.

S1.3 HILIC-FD-MS

HILIC-MS was implemented to analyze the released procainamide-labeled *N*-glycans without derivatization. In this case, 25 µL sample and 75 µL CH_3CN were added to a Waters HPLC vial followed by a 20 µL injection onto a BEH glycan 1.7 µm, 2.1 x 150 mm column (Waters Ltd., Borehamwood, UK) at 60 °C. The UHPLC was equipped with a fluorescence detector (λ_{ex} = 310 nm λ_{em} = 370 nm) with the sensitivity set to 3 and bulb power set 'long life', The separation gradient, comprised of 50 mM ammonium formate, pH 4.4 as solvent A and CH_3CN as solvent B, was performed as follows: 0 to 60 min, 76 to 51% B (flow rate of 0.4 mL/min); 60 to 64 min, 51 to 10% (flow rate 0.2 mL/min); 64 to 65 min, 10 to 76% (flow rate 0.2 mL/min); 65 to 70 min, 76 to 76% (flow rate 0.4 mL/min). The UHPLC system was coupled with an amaZon Speed ETD-ESI-MS system as described for the setup of the RPLC-FD-MS. MS and MS/MS settings were unchanged, except for a modification to the scanned mass range (*m/z* 400 – 1700).

S1.4 Intermediate precision and repeatability

The protocol repeatability was validated using full technical replicates prepared over three days (*n* = 3); intra-day 1 (*n* = 24), intra-day 2 (*n* = 13) and intra-day 3 (*n* = 13). Measurement of all replicates was performed in a single batch and the intermediate precision (*n* = 50) of the sample processing protocol could be determined.

S1.5 Fluorescence detection (FD) data processing

Fluorescent chromatograms were saved in .txt format using Chromeleon (version 7.2) and further processed using HappyTools (version 0.1-beta1, build 190115a).[2] Baseline correction was applied to all chromatograms and peak calibration was performed using the elution time of specific peaks (**Supporting Information, Table S5**). The following parameters were applied during data processing: *general*: datapoints: 100, baseline function order: 1, background window: 1; *peak detection*: peak edge type: sigma, peak edge value: 2; *calibration*: minimum number of calibrants: 4, minimum S/N: 27. Peak integration was performed using an integration window (70% FWHM) that allowed the largest peak area to be quantified while also minimizing overlap with closely eluting peaks. However, in order to support

automated quantification, a specific integration window (0.05 sec) was applied to some low abundant peaks in order to avoid integrating nearby and more abundant peaks (**Supporting Information, Table S5**). Furthermore, overlapping peaks and peaks with large shoulders were quantified using an integration window of 0.15 sec as this covered the entire peak area of both closely eluting peaks (**Supporting Information, Table S5**). Additionally, peaks were only included for further analysis if the measured retention time (RT) did not deviate more than 3 sec than the expected RT, and with a S/N above 9. Finally, the average background was determined in the blank samples by calculating the average intensity at expected peak RTs. Therefore, peaks at expected RTs were required to be above the average blank intensity plus nine times the standard deviation (s.d.) of the average blank intensity.

S1.6 MS data processing

Raw RPLC-/HILIC-MS data was converted into .mzXML files prior to being processed by LaCyTools (version 2.0, build 200723). Prior to data extraction, chromatograms were aligned according to assigned *m/z* (**Supporting Information, Table S6**) that spanned the entire RT. The alignment time window and mass window were set to 20 s and 0.1 Th, respectively. A minimum of five data points with S/N > 27 were required for alignment. Sum spectra were created for nine 'clusters' containing assigned *N*-glycans. Each sum spectra was calibrated using a mass window of 0.1 Th and based on a minimum of three calibration points (**Supporting Information, Table S6**) with S/N > 9. Finally, a minimum of 95% of the isotopic pattern was extracted for $[M+2H]^{2+}$ and $[M+3H]^{3+}$. In cases where charge states were above or below the measured mass range, no extraction was performed. Although some sodium adduct ion formation was observed (<10%), as demonstrated by **Supporting Information, Figure S5**, only fully protonated species were used for quantification.

Quality control criteria were determined and applied prior to further analysis. According to the instrument specifications, a *m/z* tolerance of ± 100 ppm was implemented. In addition, a S/N > 9 was required. Furthermore, the average isotopic pattern quality was calculated for all glycan signals with S/N > 9 for each extracted charge state. Then, an average isotopic pattern quality for all charge states was

determined for each sample and, following this, the cut-off was set to the average of all charge states plus the s.d. This resulted in an isotopic pattern quality with a deviation no greater than 35% and 20% for RPLC- and HILIC-MS data, respectively. Finally, following curation, the $[M+2H]^{2+}$ and $[M+3H]^{3+}$ signals of passing *N*-glycan compositions were summed to provide a total area of all charge states and adjusted to represent the entire isotopic envelope.

S2. Supporting Information - Results

S2.1 Method development

The investigated parameters are described in **Supporting Information, Table S2**, plasma *N*-glycans were used to perform method development, unless stated otherwise. To this end, the combination of the derivatization procedure with fluorescent labeling was examined by performing it before and after labeling. The relative abundance of the most abundant fluorescent peak was similar whether EEA was performed before or after procainamide labeling (**Supporting Information, Table S2**). In this regard, fluorescent labeling followed by EEA was selected for further processing as this format is more compatible with the current *N*-glycan analysis procedure. In this manner, *N*-glycans are initially labeled with procainamide and, as a result, there are several options for analysis as samples may be assessed using HILIC-FD-MS or further investigated using EEA and RPLC-FD-MS.

Improvements to the sensitivity and throughput of the protocol were also achieved. For example, the sensitivity of the protocol was enhanced by effectively increasing the amount of sample injected for measurement. This was performed by increasing the sample and associated reagent volumes used for the EEA reaction as well as concentrating the sample prior to EEA. Further gains in sensitivity were achieved by decreasing the run time on RPLC from 70 min to 35 min. This was obtained by increasing the gradient steepness, as well as the flow rate from 0.2 to 0.4 mL/min. Both gradients produced similar profiles (**Supporting Information, Figure S6**). However, using the 35 min gradient resulted in an increase in absolute peak areas and a decrease in peak width, whilst maintaining similar relative abundances for the ten most abundant peaks (**Supporting Information, Table S7**). A peak

corresponding to HOBt was observed in the chromatogram (**Supporting Information, Figure S6**). However, this was not detected later during method validation, possibly due to the increased precision of the sample preparation by transferring the method to the robot. Finally, sample throughput was further increased when the HILIC clean-up plate method following EEA was modified, which facilitated a plate-based processing method for EEA on the robot.

As summarized in **Supporting Information, Table S8**, the derivatization efficiency was estimated using fluorescent peak areas, which allows quantification free from ionization bias.[3,4] Ethyl esterification efficiency (α2,6 sialic acids) following fluorescent labeling was quantified using an *N*-glycan (A2G2S2 (H5N4$S_{2,6}$2)) and a human milk oligosaccharide (LST-C (H3N1$S_{2,6}$1)) as standards. Overall, this showed an efficiency of 94% (A2G2S2) and 88% (LST-C) for the expected main product of derivatization. Assigned by-products and some unassigned by-products were also detected. Assigned by-products likely arise due to some contamination with the α2,3-linkage variant or mis-derivatization (amidation) of α2,6-linked sialic acids. For the remaining (unassigned) by-products, the lack of any consistent mass shift made the source of these by-products difficult to determine. The derivatization efficiency of amidated α2,3-linked sialic acids was investigated using only the human milk oligosaccharide standard, LST-A (H3N1$S_{2,3}$1), as a complex α2,3-sialylated *N*-glycan standard was lacking. In this case, a derivatization efficiency of 91% was obtained. Similarly, assigned by-products due to under-derivatization and contamination/mis-derivatization were determined, although no unassigned by-products were observed. Despite this, no detectable amounts of expected derivatization by-products, such as under- and partial-derivatization, were determined during the examination of plasma *N*-glycans, although further unknown by-product formation was observed (**Supporting Information, Table S8**). The formation of by-products are commonly observed when employing derivatization strategies.[5,6,7] However, as detailed in the *Method Validation* section, the formation of such by-products did not affect the performance of the protocol. In the case of unknown by-product formation, future research may focus on using negative-mode ionization or orthogonal techniques such as NMR[8] in order to perform a full identification.

S3. Supporting Information – Figures

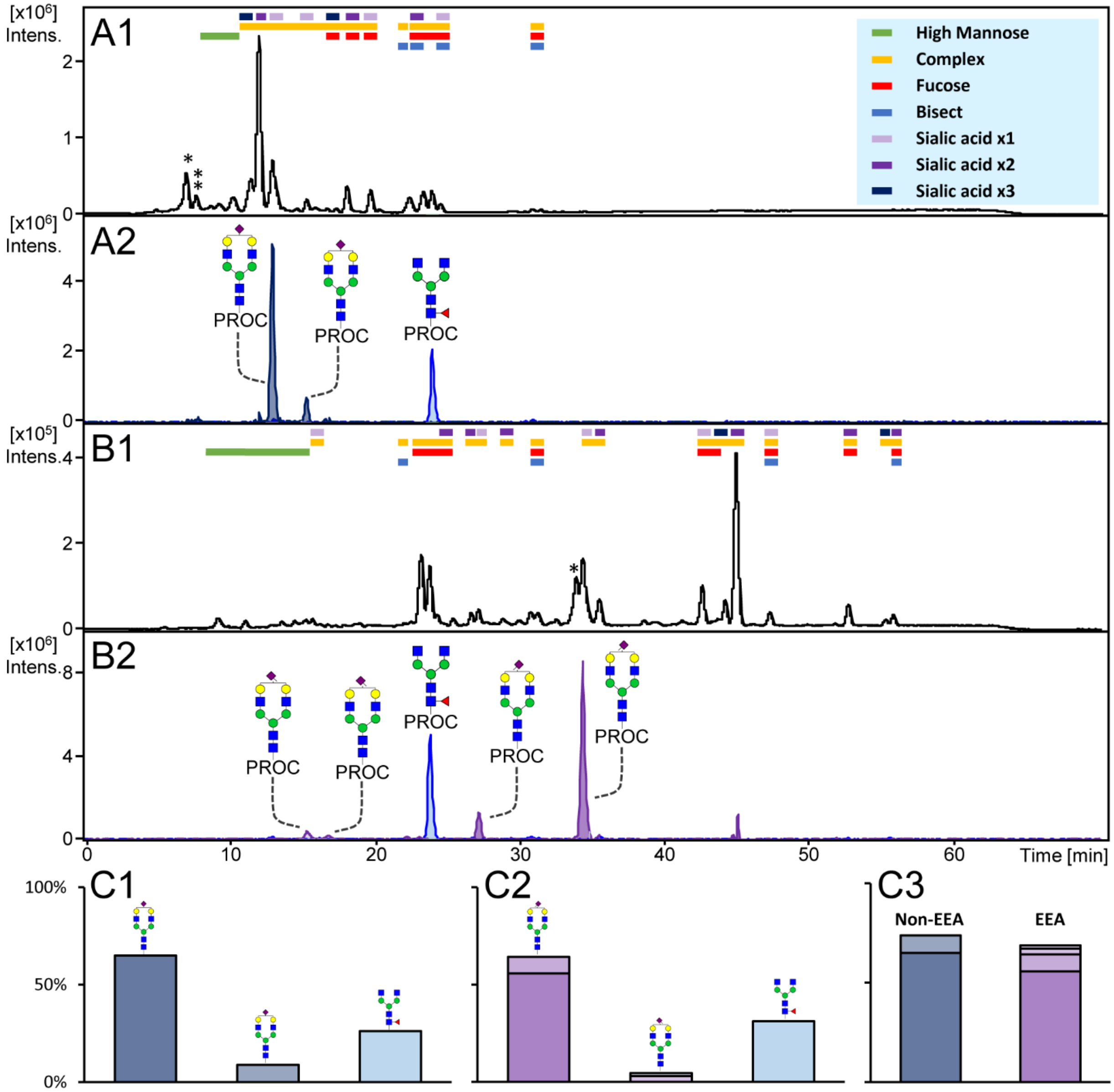

Figure S1. Non-derivatized *versus* derivatized procainamide-labeled plasma *N*-glycans on RPLC (70 min gradient). (A1) The non-derivatized profile. **(A2)** Extracted ion chromatogram of H5N4S1 (*m/z* 717.99), displaying two isomers, and H3N4F1 (*m/z* 841.90). **(B1)** The derivatized profile is provided. **(B2)** Extracted ion chromatograms show several isomers; two isomers each of H5N4S$_{2,3}$1 (*m/z* 717.63) and H5N4S$_{2,6}$1 (*m/z* 727.33). H3N4F1 is also displayed. The elution pattern is illustrated by the colored lines. Annotation is provided in the blue box. Single asterisk (*) and double asterisks (**) represent unassigned by-products of the structures H5N4S2 and H5N4S1, respectively, due to a 12 Da mass increase, likely due to a derivative of procainamide. **(C1)** The relative abundance of the peaks shown in **A2** is provided, normalized to the sum of all H5N4S1 isomers and H3N4F1; a structure unaffected by positional or sialic acid linkage isomers. **(C2)** The relative abundance of peaks illustrated in **B2** is shown (normalized to the sum of all H5N4S1 isomers and H3N4F1). **(C3)** Comparison of the relative abundance of H5N4S1 isomers between the non-derivatized and derivatized profiles normalized to the sum of H5N4S1 and H3N4F1 analytes. EEA; ethyl esterification and amidation.

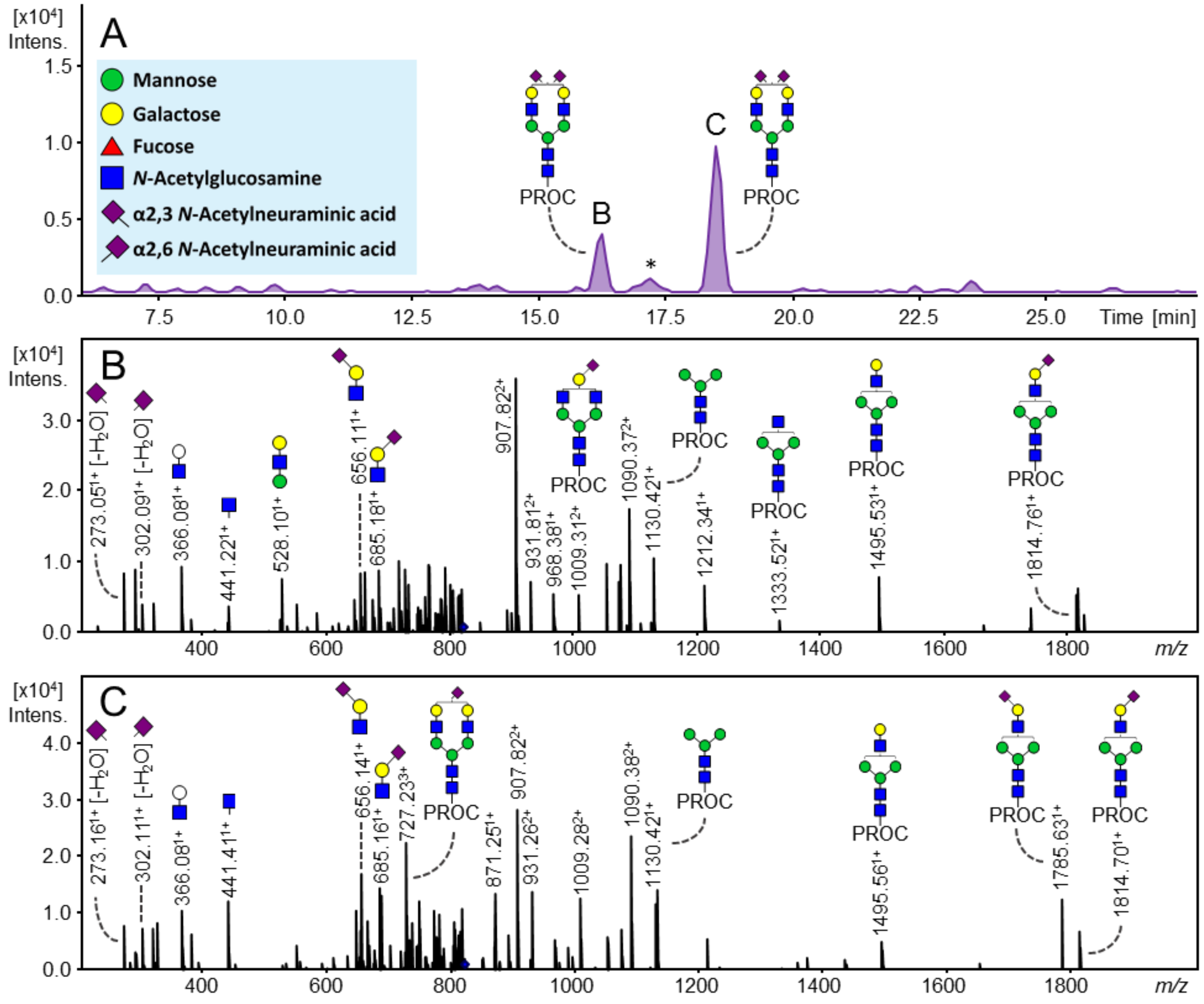

Figure S2. Sialic acid linkage-specific MS and MS/MS spectra. (A) Extracted ion chromatogram of H5N4S$_{2,3}$1S$_{2,6}$1 (*m/z* 824.01). Two peaks are illustrated as a result of separation of positional isomers. **(B)** and **(C)** MS/MS spectra of isomers 1 and 2, respectively. Specific ions such as B-ions *m/z* 656.11 and 685.18 confirm the type of sialic acid linkage(s) present, e.g. α2,3 or α2,6, respectively. In the case of multiple charge states of a single fragment, only one charge state is annotated, such as *m/z* 1814.70 $[M+H]^{1+}$ (*m/z* 907.82 $[M+2H]^{2+}$) and *m/z* 727.23 $[M+3H]^{3+}$ (**(A)** *m/z* 1090.37 and (**B**) 1090.38 $[M+2H]^{2+}$). Asterisk (*) denotes overlapping *m/z* of H5N4S$_{2,3}$1S$_{2,6}$1 with an unknown analyte (non-assigned *m/z)*. Monosaccharide annotation is provided in the blue box.

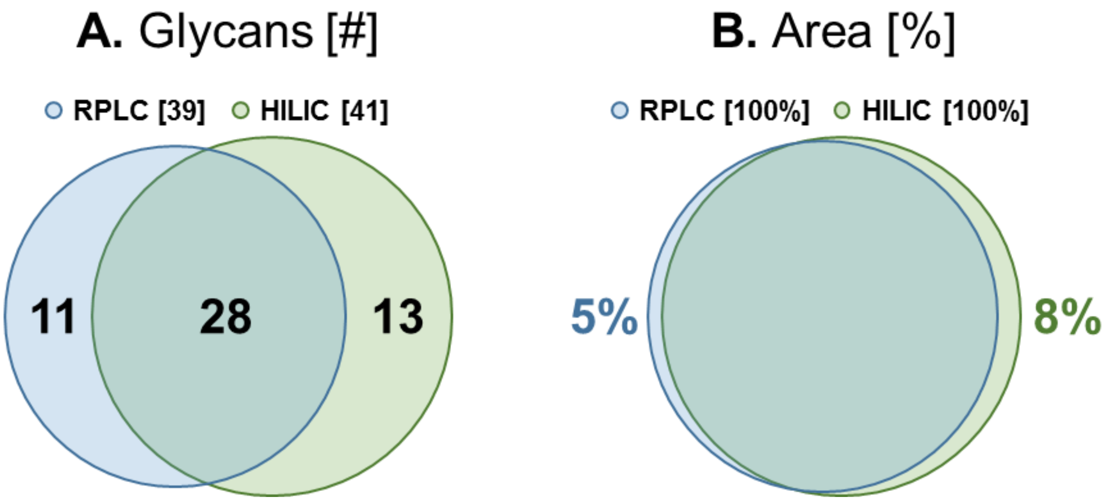

Figure S3. Unique and overlapping *N*-glycan features detected by the RPLC-FD-MS and HILIC-FD-MS platforms. (A) Number of *N*-glycans. RPLC-FD-MS (blue) detected 39 *N*-glycans in total, including 11 unique compositions. HILIC-FD-MS (green) detected 41 *N*-glycans in total, including 13 unique structures. Between both platforms, 28 overlapping *N*-glycans were detected. In some cases, one platform detected two isomers of a structure whereas the other platform detected only a single signal, which represents the sum abundance of both isomers. Thus, this was determined as one unique and one overlapping structure for the former, and one overlapping structure for the latter, respectively. **(B)** Proportion of total area [%]. RPLC-FD-MS has 5% of its total area [%] and HILIC-FD-MS has 8% of its total area [%] that does not overlap with the other platform.

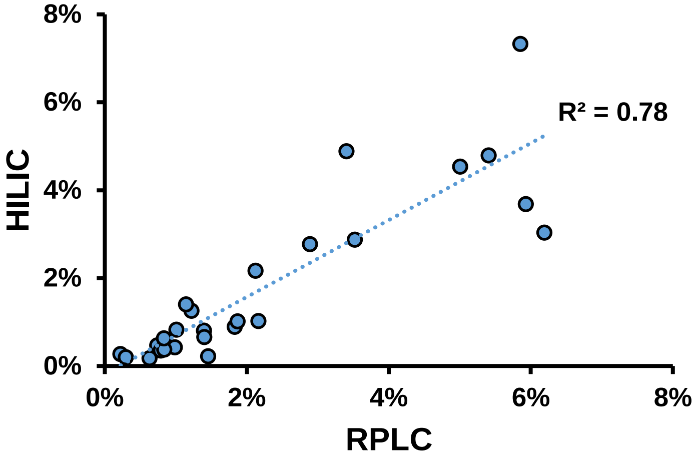

Figure S4. Comparison of relative abundances of 26 overlapping *N*-glycans detected between the RPLC-FD-MS and HILIC-FD-MS platforms. The two most abundant *N*-glycans determined by both platforms, H5N4$S_{2,6}$1 and H5N4$S_{2,6}$2 are not shown. For the purposes of performing a single comparison between *N*-glycans, several *N*-glycan isomers were summed. For HILIC-FD-MS, the relative areas of both H4N4F1 isomers were summed. For RPLC-FD-MS, isomers belonging to H7N2, H5N4$S_{2,3}$1$S_{2,6}$1 and H5N4$S_{2,6}$1 were summed. Furthermore, as H6N5S3 was quantified as the sum abundance of H6N5$S_{2,3}$1$S_{2,6}$2 and H6N5$S_{2,6}$3 by HILIC-FD-MS, these *N*-glycan abundances were also summed in RPLC-FD-MS. *N*-glycan abundances were re-normalized to the sum of the total 28 overlapping structures. The x-axis represents the relative abundances of *N*-glycans determined by RPLC-FD-MS, whereas the y-axis shows the same *N*-glycans quantified by HILIC-FD-MS. Equation of the line: $y = 0.8754x - 0.0018$. $R^2 = 0.7756$.

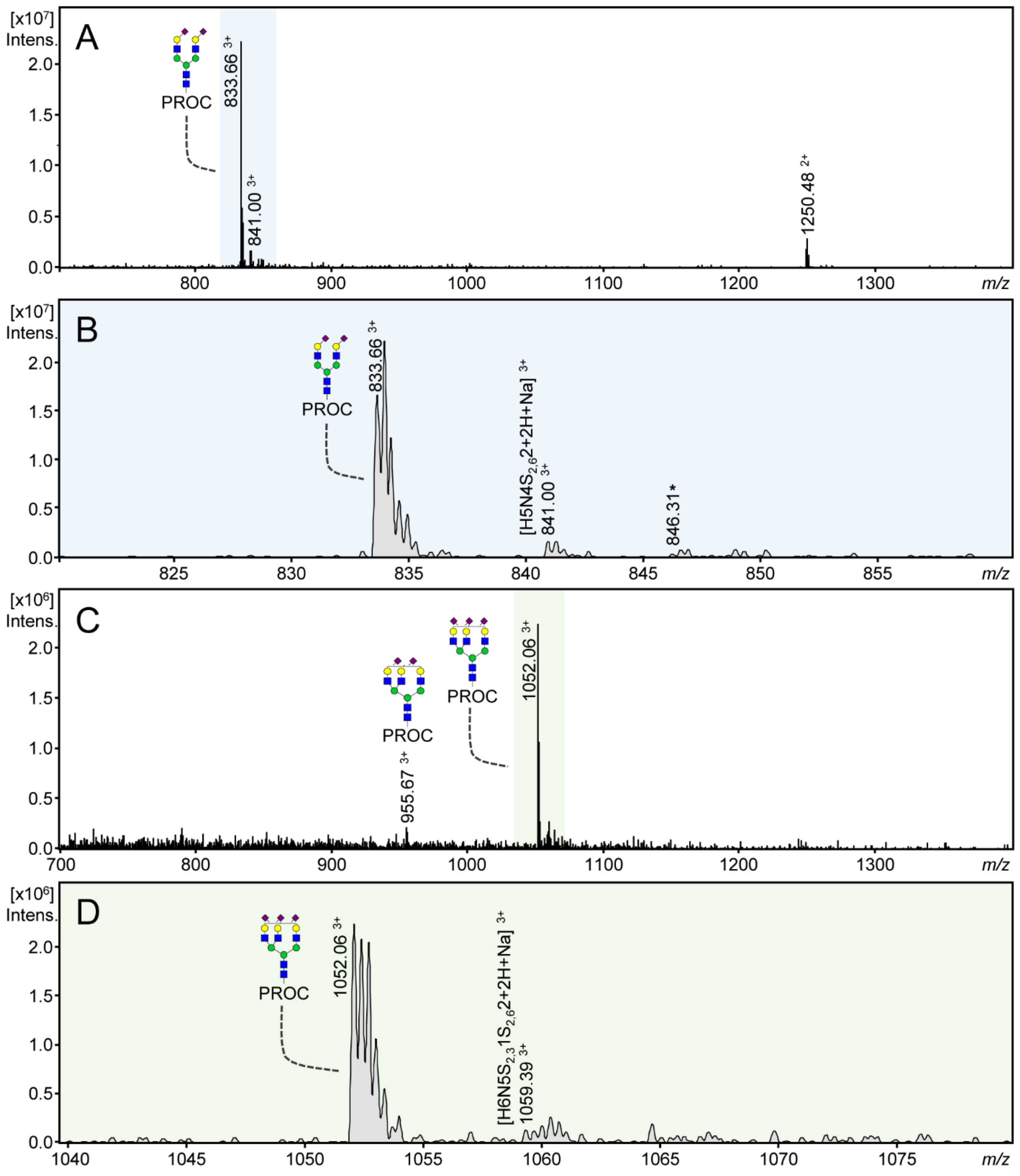

Figure S5. Adduct ion formation during MS measurement. (A) Mass spectrum of H5N4S$_{2,6}$2 $[M+2H]^{2+}$ (*m/z* 1250.48) and $[M+3H]^{3+}$ (*m/z* 833.66). **(B)** Mass spectrum of H5N4S$_{2,6}$2 $[M+3H]^{3+}$ (*m/z* 833.66). The adduct ion $[M + 2H + Na]^{3+}$ (*m/z* 841.00) is also displayed with a relative abundance of 10% in comparison with the main protonated species. **(C)** Mass spectrum of H6N5S$_{2,3}$1S$_{2,6}$2 $[M+3H]^{3+}$ (*m/z* 1052.66). H6N5S$_{2,6}$2 $[M+3H]^{3+}$ (*m/z* 955.67) was also detected. **(D)** Mass spectrum of H6N5S$_{2,3}$1S$_{2,6}$2 $[M+3H]^{3+}$ (*m/z* 1052.66). The adduct ion $[M + 2H + Na]^{3+}$ (*m/z* 1059.39) is shown with a relative abundance of 6% in comparison with the main protonated species. Asterisk (*) denotes an unassigned *m/z*.

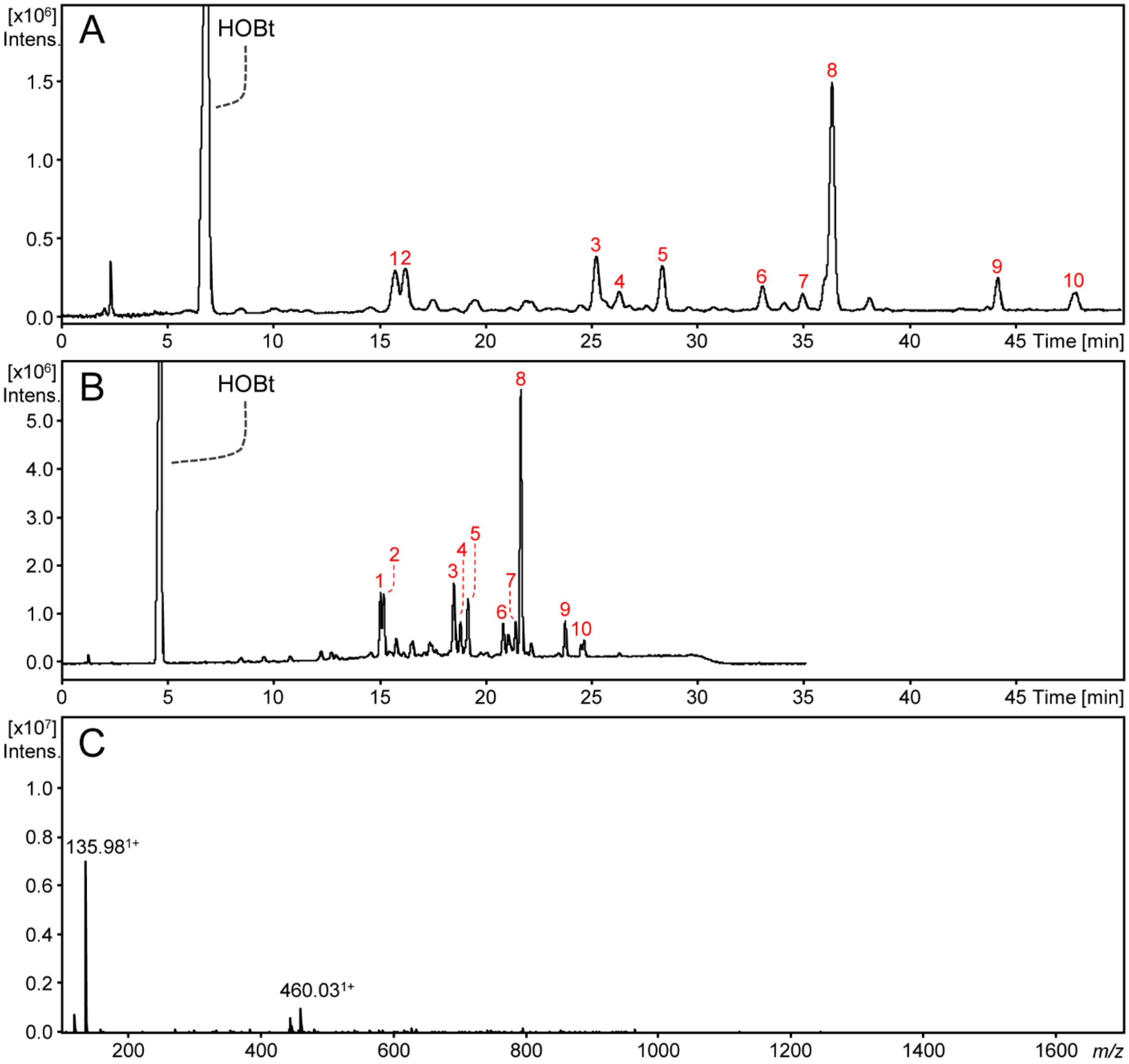

Figure S6. Derivatized and procainamide-labeled plasma *N*-glycan profile on RPLC. Solvent A (50 mM ammonium formate) and solvent B (10% CH3CN; 0.1% FA (v/v) were used for both gradients. **(A) 70 min gradient.** 0 to 53.5 min, 40 to 80% B; 53.5 to 57.5 min, 80% B; 57.5 to 59.5 min, 80 to 40% B; 59.5 to 70.1 min, 40% B; 0.2 mL/min. **(B) 35 min gradient.** 0 to 26.5 min, 15 to 95% B; 26.5 to 28.5 min, 95% B; 28.5 to 29.5 min, 95 to 15% B; 29.5 to 35.1 min, 15% B; 0.4 mL/min. Numbered peaks (10 most abundant peaks) in red were used for the purposes of comparison, as shown in **Supporting Information, Table S7**. **(C) MS spectrum of HOBt peak.** Mass spectrum of the peak corresponding to HOBt, with a theoretical *m/z* of *m/z* 136.05 $[M+H]^{1+}$.

S4. References

(1) Kotsias, M.; Blanas, A.; van Vliet, S. J.; Pirro, M.; Spencer, D. I. R.; Kozak, R. P. Method comparison for N-glycan profiling: Towards the standardization of glycoanalytical technologies for cell line analysis. *PLoS One* **2019**, *14* (10), e0223270. DOI: 10.1371/journal.pone.0223270.

(2) Jansen, B. C.; Hafkenscheid, L.; Bondt, A.; Gardner, R. A.; Hendel, J. L.; Wuhrer, M.; Spencer, D. I. R. HappyTools: A software for high-throughput HPLC data processing and quantitation. *PLoS One* **2018**, *13* (7), e0200280. DOI: 10.1371/journal.pone.0200280.

(3) de Haan, N.; Yang, S.; Cipollo, J.; Wuhrer, M. Glycomics studies using sialic acid derivatization and mass spectrometry. *Nature Reviews Chemistry* **2020**, *4* (5), 229-242. DOI: 10.1038/s41570-020-0174-3.

(4) Suzuki, N.; Abe, T.; Natsuka, S. Quantitative LC-MS and MS/MS analysis of sialylated glycans modified by linkage-specific alkylamidation. *Analytical Biochemistry* **2019**, *567*, 117-127. DOI: 10.1016/j.ab.2018.11.014.

(5) Holst, S.; Heijs, B.; de Haan, N.; van Zeijl, R. J.; Briaire-de Bruijn, I. H.; van Pelt, G. W.; Mehta, A. S.; Angel, P. M.; Mesker, W. E.; Tollenaar, R. A.; Drake, R. R.; Bovee, J. V.; McDonnell, L. A.; Wuhrer, M. Linkage-Specific in Situ Sialic Acid Derivatization for N-Glycan Mass Spectrometry Imaging of Formalin-Fixed Paraffin-Embedded Tissues. *Anal Chem* **2016**, *88* (11), 5904-5913. DOI: 10.1021/acs.analchem.6b00819.

(6) Nishikaze, T.; Tsumoto, H.; Sekiya, S.; Iwamoto, S.; Miura, Y.; Tanaka, K. Differentiation of Sialyl Linkage Isomers by One-Pot Sialic Acid Derivatization for Mass Spectrometry-Based Glycan Profiling. *Analytical Chemistry* **2017**, *89* (4), 2353-2360. DOI: 10.1021/acs.analchem.6b04150.

(7) Tousi, F.; Bones, J.; Hancock, W. S.; Hincapie, M. Differential chemical derivatization integrated with chromatographic separation for analysis of isomeric sialylated N-glycans: a nano-hydrophilic interaction liquid chromatography-MS platform. *Anal Chem* **2013**, *85* (17), 8421-8428. DOI: 10.1021/ac4018007.

(8) Pongracz, T.; Verhoeven, A.; Wuhrer, M.; de Haan, N. The structure and role of lactone intermediates in linkage-specific sialic acid derivatization reactions. *Glycoconj J* **2021**, *38* (2), 157-166. DOI: 10.1007/s10719-020-09971-7.
